# Mental Health Risk Factors for Postoperative Pain in Head and Neck Free Flap Reconstruction Patients

**DOI:** 10.1002/ohn.70269

**Published:** 2026-04-30

**Authors:** Daniel R. S. Habib, Sindhura Sridhar, Heezy Suh, Daniel Larson, Brooke B. Swain, Kelly Vittetoe, Donald Sengstack, Michael C. Topf, Melanie D. Hicks

**Affiliations:** ^1^ Vanderbilt University School of Medicine Nashville Tennessee USA; ^2^ Department of Otolaryngology–Head and Neck Surgery Vanderbilt University Medical Center Nashville Tennessee USA; ^3^ Department of Biomedical Informatics Vanderbilt University Medical Center Nashville Tennessee USA

**Keywords:** anxiety disorder, depressive disorder, free flap, head and neck cancer, mental health, postoperative pain

## Abstract

**Objective:**

Head and neck cancer (HNC) surgery is associated with postoperative pain and comorbid mental health disorders (MHDs). This study assessed predictors of postoperative pain and inpatient pain service consultation following free flap reconstruction (FFR).

**Study Design:**

Retrospective cohort.

**Setting:**

Single‐institution database.

**Methods:**

Multivariable linear and logistic regressions identified predictors of postoperative day 5 (POD5) pain scores (0‐10) and pain consultation, respectively. Propensity‐matched analyses compared pain trajectories by MHD (depression or anxiety) and pain consultation.

**Results:**

The cohort included 645 FFR patients. Higher POD5 pain was associated with MHD (*β*: 0.43; 95% CI: 0.03, 0.84), currently smoking (*β*: 0.58; 95% CI: 0.14, 1.03), being separated/divorced versus single (*β*: 0.71; 95% CI: 0.03, 1.39), osteocutaneous radial forearm free flap (OCRFFF) versus fasciocutaneous radial forearm free flap (RFFF) (*β*: 0.54; 95% CI: 0.02, 1.05), laryngeal site versus oral cavity (*β*: 0.59; 95% CI: 0.06, 1.13; *P* = .029), and non‐oncologic indication (*β*: 0.86; 95% CI: 0.06, 1.67; *P* = .036). Pain consultation was associated with MHD (adjusted odds ratio [aOR]: 1.99; 95% CI: 1.13‐3.52), preoperative narcotic use (aOR: 3.20; 95% CI: 1.77‐5.79), OCRFFF (aOR: 3.42; 95% CI: 1.53‐7.66), and private insurance (aOR: 3.09; 95% CI: 1.46‐6.58). Among propensity‐matched patients with an MHD, pain scores were not significantly different by pain consultation, while non‐MHD patients with pain consultation reported higher pain across POD1‐5 (*P* < .01).

**Conclusion:**

Psychosocial and clinical factors such as MHD diagnosis were independently associated with pain scores and pain service consultation after FFR. Patients with an MHD are particularly vulnerable. Identifying risk factors preoperatively may guide personalized perioperative pain management strategies.

Free flap reconstruction (FFR) surgery was established as the primary method of reconstruction for head and neck cancer (HNC) in the 1990s.^1^ Despite declining postoperative mortality rates^2^ and advances in multimodal pain management, postoperative pain continues to be a significant clinical concern. In other settings, having no established pain plan is associated with longer hospital stay, higher outpatient opioid quantities, and increased resource utilization.^3^ HNC patients report higher rates of pain after curative treatment than the general cancer patient population: 45%^4^, ^5^ compared to 35.8%, respectively.^6^ Post‐treatment pain has been associated with lower quality of life, chronic opioid use, and major depression.^5^, ^7^ The association between pain and psychiatric comorbidities is especially relevant, as 35% of HNC FFR patients have moderate to severe depression,^8^ and the prevalence of depression continues to increase in HNC patients.^9^ Depression has also been shown to affect long‐term physical function^10^ and rates of rehospitalization after surgery in HNC patients.^9^ These data suggest a strong association between MHD, sociodemographics, and postoperative pain in surgical cancer patients, but how they affect FFR patients with HNC remains unknown.

Managing postoperative pain in HNC is challenging, as many of the opioid‐prescribing guidelines primarily address non‐malignant pain and therefore often exclude cancer‐related postoperative pain.^11^, ^12^ Existing studies have shown that younger patient age, comorbid anxiety, moderate to severe preoperative pain (on validated pain assessment instruments), and bony free flaps are associated with higher postoperative pain scores after FFR.^13^, ^14^ Additionally, lower socioeconomic status and black race have been associated with a higher risk of postoperative complications in HNC patients.^15^, ^16^ In contrast, social support and marriage have been shown to predict better survival and quality of life among patients with HNC.^17^, ^18^ Although the association between several HNC patient factors and broader outcomes has been studied, the literature lacks a comprehensive assessment of the association between patient characteristics and postoperative pain scores in patients with MHD who have HNC and have undergone FFR. Additionally, there are no studies in this patient population that investigate inpatient pain service consultation, which may indicate inadequately controlled pain and complex pain management needs.^19^

This study aims to address these gaps by analyzing a cohort of patients who underwent HNC FFR at a high‐volume tertiary care institution. This study assesses the relationships between demographic, psychosocial, and surgical factors and postoperative pain outcomes (ie, average POD5 pain score and pain service consultation). By identifying preoperative risk factors for higher postoperative pain, this study seeks to inform individualized postoperative pain management and improve long‐term patient outcomes.

## Methods

### Study Design and Population

This was a single‐institution retrospective cohort study. Adult patients undergoing head and neck FFR from January 1, 2019, to December 31, 2023, were included. Patients were included if their medical record was associated with both an FFR Current Procedural Terminology (CPT) code and a neck dissection or exploration CPT code as an indicator of vessel identification (Supplemental Table S1, available online). Patients were included if they underwent FFR for oncologic (ie, primary malignancy or recurrence) as well as other indications (eg, radionecrosis, fistula repair, osteomyelitis, dehiscence, plate exposure, prior defects, afunctional larynx, and wound complications). Patients with missing data or an FFR indication of a gunshot wound were excluded.

Patient demographic data, comorbidities, oncologic history, psychiatric diagnoses, medications, and surgical details were collected via chart review after reaching consensus about important variables to include and where to find information within each chart. Mental health disorder (MHD) was defined as having a depression or anxiety diagnosis, the two most prevalent conditions in this population, by International Classification of Diseases (ICD)‐9 and ‐10 codes (Supplemental Table S2, available online). Other psychiatric diagnoses such as bipolar disorder or posttraumatic stress disorder (PTSD) were not included to maintain a focused definition of MHD, avoiding the added heterogeneity that may arise from less common or more severe conditions. This study was approved by the Vanderbilt University Medical Center Institutional Review Board (#241879) and was conducted in accordance with the Strengthening the Reporting of Observational Studies in Epidemiology (STROBE) reporting guidelines (Supplemental Table S3, available online).

### Outcomes and Statistical Analysis

We performed descriptive analyses reporting continuous variables as medians (interquartile ranges, IQRs) and categorical variables as frequencies (percentages). We performed Wilcoxon rank‐sum tests for continuous variables and Pearson's chi‐square test or Fisher's exact test for categorical variables. Outcome measures included postoperative day 5 (POD5) pain score and postoperative pain service consultation. Pain score was a score reported by patients and charted by nurses every 4 hours during their inpatient stay as part of routine care on a scale of 0 to 10, with 10 being the most pain. These data were directly documented from the electronic medical record. Linear and logistic regressions were performed for POD5 pain score and pain service consultation, respectively, to assess their associations with patient, tumor, and reconstruction characteristics. Both univariable and multivariable analyses were performed. Covariates included age at time of surgery, sex, race, marital status, health insurance type, current smoking status, current alcohol use, Carlson‐Deyo Comorbidity Index (CDCI), FF site, FF type, indication for surgery, MHD, preoperative narcotic prescription, and psychiatry consult. Univariable and multivariable regressions were also performed separately for oncologic cases; regressions were inappropriate for non‐oncologic cases due to the small sample size and were therefore not performed. To compare average POD1‐5 pain scores, propensity matching of patients by MHD diagnosis was performed at a 1:1 ratio with a caliper of 0.15, controlling for the same covariates used in the multivariable regressions. The same propensity score matching approach was also performed two more times by pain consultation among patients (1) with and (2) without an MHD diagnosis to assess the relationship of pain consultation on POD1‐5 pain scores. Two‐sample *t* tests were performed to compare POD1‐5 pain scores across groups. We performed all statistical analyses using R statistical software version 4.3.3. Significance was set a priori at *P* < .05.

## Results

Of 709 patients extracted by CPT codes, the final cohort included 645 FF patients (Table 1). Patients were predominantly white (91.6%) and male (69.3%), with a median age of 65 years (IQR 57‐72). The most common FF types were fasciocutaneous radial forearm (RFFF, 50.4%), osteocutaneous radial forearm (OCRFFF, 18.9%), and anterolateral thigh (ALT, 16.6%) flaps. Overall, 41.2% of patients had a preoperative narcotic prescription, and 13.2% received an inpatient pain consult. When stratified by MHD, patients with MHD (n = 222) were younger (median [IQR] age 63 [56‐70] vs 66 [58‐72], *P* = .005), and more likely to be female (40.5% vs 25.5%, *P* < .001) and white (95.9% vs 89.4%, *P* = .008) compared to patients without MHD. The MHD group was also more likely to have a preoperative narcotic prescription (50.5% vs 36.4%, *P* < .001), and to receive an inpatient pain consult (21.2% vs 9.0%, *P* < .001) or psychiatric consult (7.7% vs 3.5%, *P* = .022). There were no significant differences between groups in surgical indication, comorbidity burden, flap site, flap type, or pathologic stage.

**Table 1:** Cohort Characteristics by Mental Health Disorder^a^

Variable	All, count (%) [N = 645]	MHD, count (%) [N = 222]	No MHD, count (%) [N = 423]	*P* value
Age at time of surgery, median [IQR]	65 [57‐72]	63 [56‐70]	66 [58‐72]	**.005**
Sex				**<.001**
Female	198 (30.7%)	90 (40.5%)	108 (25.5%)	
Male	447 (69.3%)	132 (59.5%)	315 (74.5%)	
Race				**.008**
White	591 (91.6%)	213 (95.9%)	378 (89.4%)	
Not white	43 (6.7%)	7 (4.1%)	36 (10.6%)	
Marital status				.236
Single	126 (19.5%)	45 (20.3%)	81 (19.1%)	
Married/significant other	377 (58.4%)	122 (55.0%)	255 (60.3%)	
Separated/divorced	75 (11.6%)	33 (14.9%)	42 (9.9%)	
Widowed	59 (9.1%)	18 (8.1%)	41 (9.7%)	
Insurance type				.091
Private	197 (30.5%)	64 (28.8%)	133 (31.4%)	
Medicare or other government	431 (66.8%)	156 (70.3%)	275 (65%)	
Medicaid or none	17 (2.6%)	2 (0.9%)	15 (3.5%)	
Current smoking status	177 (27.4%)	61 (27.5%)	116 (27.4%)	.988
Current alcohol use	193 (29.9%)	48 (21.6%)	145 (34.3%)	**<.001**
CDCI, median [IQR]	4 [3‐6]	4.5 [3‐6]	4 [3‐6]	.919
Site				.167
Oral cavity	387 (60%)	133 (59.9%)	254 (60%)	
Oropharynx	30 (4.7%)	9 (4.1%)	21 (5%)	
Larynx	108 (16.7%)	48 (21.6%)	60 (14.2%)	
Sinonasal	22 (3.4%)	5 (2.3%)	17 (4%)	
Cutaneous	82 (12.7%)	26 (11.7%)	56 (13.2%)	
Pathologic T stage				.403
pT1	30 (4.7%)	15 (6.8%)	15 (3.5%)	
pT2	67 (10.4%)	23 (10.4%)	44 (10.4%)	
pT3	110 (17.1%)	37 (16.7%)	73 (17.3%)	
pT4	262 (40.6%)	93 (41.9%)	169 (40%)	
Pathologic N stage				.450
pN0	245 (38%)	93 (41.9%)	152 (35.9%)	
pN1	56 (8.7%)	15 (6.8%)	41 (9.7%)	
pN2	65 (10.1%)	22 (9.9%)	43 (10.2%)	
pN3	79 (12.2%)	27 (12.2%)	52 (12.3%)	
Free flap type				.376
Radial forearm	325 (50.4%)	119 (53.6%)	206 (48.7%)	
Osteocutaneous radial forearm	122 (18.9%)	46 (20.7%)	76 (18%)	
Anterolateral thigh	107 (16.6%)	30 (13.5%)	77 (18.2%)	
Fibula	28 (4.3%)	9 (4.1%)	19 (4.5%)	
Other/multiple	63 (9.8%)	18 (8.1%)	45 (10.6%)	
Indication				.736
Primary malignancy	391 (60.6%)	130 (58.6%)	261 (61.7%)	
Recurrence	209 (32.4%)	76 (34.2%)	133 (31.4%)	
Non‐oncologic	45 (7%)	16 (7.2%)	29 (6.9%)	
Depression	158 (24.5%)	158 (71.2%)	0 (0%)	NA
Anxiety	160 (24.8%)	160 (72.1%)	0 (0%)	NA
Preoperative narcotic prescription	266 (41.2%)	112 (50.5%)	154 (36.4%)	**<.001**
Inpatient psychiatry consult	32 (5%)	17 (7.7%)	15 (3.5%)	**.022**
Inpatient pain consult	85 (13.2%)	47 (21.2%)	38 (9%)	**<.001**

Bold values are statistically significant at *P* values.

Abbreviations: CDCI, Charlson‐Deyo Comorbidity Index; IQR, interquartile range; MHD, mental health disorder.

^a^ Percentages might not sum to 100% due to unavailable data. Other/multiple flap type encompasses ulnar, serratus, scapular, medial sural, or any combination of two or more of the listed flaps.

There were significant associations between POD5 pain scores and patient characteristics (Table 2). On multivariable linear regression of pain scores (0‐10), lower POD5 pain was associated with older age (*β*: −0.06; 95% CI: −0.08, −0.04; *P* < .001). In contrast, higher POD5 pain was associated with being separated or divorced compared to single patients (*β*: 0.71; 95% CI: 0.03, 1.39; *P* = .041), currently smoking (*β*: 0.58; 95% CI: 0.14, 1.03; *P* = .011), laryngeal resection site compared to oral cavity (*β*: 0.59; 95% CI: 0.06, 1.13; *P* = .029), OCRFF (*β*: 0.54; 95% CI: 0.02, 1.05; *P* = .041) compared to fasciocutaneous RFFF, non‐oncologic indication compared with primary malignancy (*β*: 0.86; 95% CI: 0.06, 1.67; *P* = .036), and MHD (*β*: 0.43; 95% CI: 0.03, 0.84; *P* = .037). There was a nonsignificant trend toward higher POD5 pain with ALT flap compared to fasciocutaneous RFFF (*β*: 0.57; 95% CI: −0.02, 1.16; *P* = .061) and preoperative narcotic prescription (*β*: 0.37; 95% CI: −0.03, 0.76; *P* = .069). There were no significant associations between POD5 pain and sex, race, insurance, alcohol use, CDCI, and psychiatry consult. After excluding non‐oncologic cases, higher pain score associations held for lower age, current smoking status, and MHD but not for separated/divorced status and laryngeal resection site (Supplemental Table S4, available online). Although the association with OCRFFF was no longer significant (*β*: 0.51; 95% CI: −0.02, 1.03; *P* = .060), it was significant for ALT flap compared to fasciocutaneous RFFF (*β*: 0.63; 95% CI: 0.02, 1.23; *P* = .043).

**Table 2:** Univariable and Multivariable Linear Regressions of Patient and Tumor Characteristics by Postoperative Day 5 Pain Score^a^

Variable	Univariable		Multivariable	
	*β* (95% CI)	*P* value	*β* (95% CI)	*P* value
Age at time of surgery	−0.06 (−0.08, −0.05)	**<.001**	−0.06 (−0.08, −0.04)	**<.001**
Female sex (vs male)	−0.06 (−0.46, 0.34)	.771	0.01 (−0.42, 0.43)	.978
Non‐white race (vs white)	−0.43 (−1.15, 0.29)	.246	−0.50 (−1.22, 0.21)	.169
Marital status				
Single	0.39 (−0.08, 0.86)	.107	Reference	NA
Married/significant other	−0.39 (−0.77, −0.01)	**.047**	−0.05 (−0.55, 0.45)	.853
Separated/divorced	0.98 (0.40, 1.56)	**.001**	0.71 (0.03, 1.39)	**.041**
Widowed	−0.83 (−1.48, −0.19)	**.011**	0.04 (−0.75, 0.82)	.930
Private insurance (vs not private)	−0.44 (−0.85, −0.03)	**.035**	0.26 (−0.21, 0.74)	.276
Current smoking status (vs not)	1.02 (0.6, 1.45)	**<.001**	0.58 (0.14, 1.03)	**.011**
Current alcohol use (vs not)	0.19 (−0.23, 0.61)	.387	−0.04 (−0.46, 0.38)	.859
CDCI	−0.14 (−0.22, −0.07)	**<.001**	−0.01 (−0.10, 0.07)	.777
Site				
Oral cavity	−0.09 (−0.49, 0.32)	.676	Reference	NA
Oropharynx	−0.39 (−1.23, 0.45)	.368	−0.09 (−0.99, 0.80)	.838
Larynx	0.65 (0.17, 1.13)	**.008**	0.59 (0.06, 1.13)	**.029**
Sinonasal/cutaneous	−0.74 (−1.39, −0.08)	**.028**	−0.22 (−0.91, 0.48)	.541
Free flap type				
Radial forearm	−0.27 (−0.65, 0.11)	.162	Reference	NA
Osteocutaneous radial forearm	0.01 (−0.44, 0.47)	.949	0.54 (0.02, 1.05)	**.041**
Anterolateral thigh	0.24 (−0.30, 0.78)	.380	0.57 (−0.02, 1.16)	.061
Fibula	0.57 (−0.33, 1.48)	.212	0.66 (−0.25, 1.57)	.157
Other/multiple	0.11 (−0.52, 0.74)	.733	0.14 (−0.52, 0.80)	.678
Indication				
Primary malignancy	−0.17 (−0.56, 0.22)	.403	Reference	NA
Recurrence	−0.08 (−0.49, 0.33)	.707	0.01 (−0.44, 0.46)	.955
Non‐oncologic	0.94 (0.17, 1.71)	**.017**	0.86 (0.06, 1.67)	**.036**
MHD	0.75 (0.37, 1.14)	**<.001**	0.43 (0.03, 0.84)	**.037**
Pre‐op narcotic prescription	0.78 (0.40, 1.16)	**<.001**	0.37 (−0.03, 0.76)	.069
Psychiatry consult	0.42 (−0.41, 1.25)	.320	−0.16 (−0.97, 0.66)	.707

Bold values are statistically significant at *P* values.

Abbreviations: *β*, coefficient resulting from linear regression; CDCI, Charlson‐Deyo Comorbidity Index; CI, confidence interval; MHD, mental health disorder; NA, not applicable.

^a^ Other/multiple flap type encompasses ulnar, serratus, scapular, medial sural, or any combination of two or more of the listed flaps.

On multivariable logistic regression, several factors were independently associated with receiving an inpatient pain service consultation (Table 3). Higher odds of pain consult were observed among patients with an MHD (adjusted odds ratio [aOR]: 1.99; 95% CI: 1.13, 3.52; *P* = .018), patients with a preoperative narcotic prescription (aOR: 3.20; 95% CI: 1.77, 5.79; *P* < .001), and patients with private insurance (aOR: 3.09; 95% CI: 1.46, 6.58; *P* = .003). Patients reconstructed with an OCRFFF also had higher odds of pain consultation than patients with fasciocutaneous RFFF (aOR: 3.42; 95% CI: 1.53, 7.66; *P* = .003). In contrast, older age was associated with lower odds of pain consultation (aOR: 0.95; 95% CI: 0.92, 0.98; *P* = .001). There was a nonsignificant trend toward higher odds of pain consultation with the laryngeal resection site compared with the oral cavity (aOR: 2.04; 95% CI: 0.97, 4.31; *P* = .060). There were no significant associations between inpatient pain consultation and sex, race, marital status, smoking, alcohol use, flap indication, or inpatient psychiatry consult. After excluding non‐oncologic cases, pain consultation associations held for lower age, private insurance, OCRFFF, MHD, and preoperative narcotic prescription (Supplemental Table S5, available online). Additionally, pain consultation after oncologic cases was also associated with lower CDCI (aOR: 0.84; 95% CI: 0.72, 0.98; *P* = .027).

**Table 3:** Univariable and Multivariable Logistic Regression of Patient and Tumor Characteristics by Postoperative Pain Service Consultation^a^

Variable	Univariable		Multivariable	
	OR (95% CI)	*P* value	aOR (95% CI)	*P* value
Age at time of surgery	0.96 (0.94, 0.98)	**<.001**	0.95 (0.92, 0.98)	**.001**
Female sex (vs male)	1.13 (0.69, 1.83)	.630	0.97 (0.52, 1.80)	.914
Non‐white race (vs white)	1.05 (0.43, 2.57)	.913	0.62 (0.19, 2.09)	.443
Marital status				
Single	1.77 (1.05, 2.97)	**.032**	Reference	NA
Married/significant other	0.55 (0.35, 0.87)	**.011**	0.89 (0.44, 1.79)	.741
Separated/divorced	1.79 (0.96, 3.32)	.066	1.37 (0.57, 3.30)	.476
Widowed	0.73 (0.30, 1.74)	.474	1.02 (0.32, 3.27)	.972
Private insurance (vs not private)	1.50 (0.88, 2.56)	.134	3.09 (1.46, 6.58)	**.003**
Current smoking status (vs not)	1.27 (0.77, 2.07)	.349	1.06 (0.56, 1.99)	.857
Current alcohol use (vs not)	0.78 (0.46, 1.32)	.354	0.72 (0.37, 1.38)	.317
CDCI	0.88 (0.79, 0.97)	**.015**	0.90 (0.79, 1.02)	.101
Site				
Oral cavity	0.94 (0.59, 1.51)	.796	Reference	NA
Oropharynx	0.72 (0.21, 2.43)	.598	2.65 (0.64, 11.01)	.180
Larynx	1.93 (1.13, 3.31)	**.017**	2.04 (0.97, 4.31)	.060
Sinonasal/cutaneous	0.50 (0.23, 1.07)	.074	0.53 (0.19, 1.50)	.232
Free flap type				
Radial forearm	0.65 (0.41, 1.04)	.070	Reference	NA
Osteocutaneous radial forearm	1.38 (0.8, 2.38)	.245	3.42 (1.53, 7.66)	**.003**
Anterolateral thigh	0.80 (0.42, 1.54)	.512	1.38 (0.57, 3.32)	.477
Fibula	1.46 (0.54, 3.95)	.457	1.80 (0.49, 6.65)	.380
Other/multiple	1.84 (0.95, 3.56)	.069	2.05 (0.84, 4.97)	.113
Indication				
Primary malignancy	0.87 (0.55, 1.38)	.547	Reference	NA
Recurrence	0.85 (0.52, 1.4)	.527	0.63 (0.32, 1.22)	.169
Non‐oncologic	2.30 (1.12, 4.73)	**.024**	2.15 (0.83, 5.57)	.117
MHD	2.72 (1.71, 4.33)	**<.001**	1.99 (1.13, 3.52)	**.018**
Pre‐op narcotic prescription	3.42 (2.11, 5.55)	**<.001**	3.20 (1.77, 5.79)	**<.001**
Psychiatry consult	2.32 (1.01, 5.35)	**.048**	1.25 (0.44, 3.57)	.679

Bold values are statistically significant at *P* values.

Abbreviations: aOR, adjusted odds ratio; CDCI, Charlson‐Deyo Comorbidity Index; CI, confidence interval; MHD, mental health disorder; NA, not applicable; OR, odds ratio.

^a^ Other/multiple flap type encompasses ulnar, serratus, scapular, medial sural, or any combination of two or more of the listed flaps.

**Figure 1:**
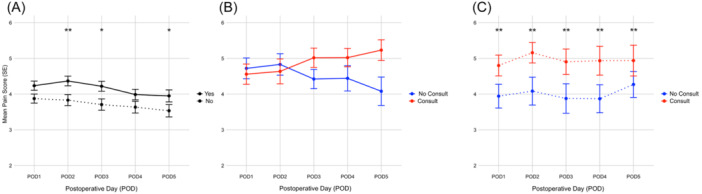
Average postoperative pain scores by (A) presence of mental health disorder (MHD) and by postoperative pain consult (B) with MHD and (C) without MHD. *Note*: Solid line depicts the presence of MHD, while the dotted line depicts the absence of MHD. The red line depicts the presence of inpatient pain consultation, while the blue line depicts the absence of inpatient pain consultation. *P < .05, **P < .01.

Figure 1 illustrates average postoperative pain scores by MHD and pain consultation after matching patients by the same covariates used in the multivariable regressions. Among 396 matched patients, patients with MHD reported significantly higher pain scores than patients without MHD on POD2 (*P* = .005), POD3 (*P* = .030), and POD5 (*P* = .029). Among 62 matched patients without MHD, pain consultation was associated with significantly higher pain scores on all postoperative days (all *P* < .01). In contrast, among 70 matched patients with MHD, pain scores did not differ significantly by pain consultation.

## Discussion

In this retrospective study of 645 FF patients, both demographic and clinical factors were associated with postoperative pain score and pain service utilization. Younger age, separated or divorced marital status, current smoking status, laryngeal site, OCRFFF, non‐oncologic indication, and MHD were independently associated with higher POD5 pain scores. Inpatient pain consults were more likely among patients with younger age, private insurance, OCRFFF, MHD, and preoperative narcotic prescription. These findings suggest that specific patient, tumor, and treatment characteristics can help clinicians identify individuals at risk for higher pain burden and guide preoperative counseling and perioperative pain management strategies.

Previous studies have identified predictors of increased postoperative pain following HNC FFR, including younger age, bony flap reconstruction, and comorbid mental health conditions (in a smaller cohort with fewer covariates).^13^, ^14^, ^20^ Our findings confirm these associations in a larger cohort of FFR patients. Notably, we observed that current smoking status was independently associated with higher pain scores. This was not surprising given that patients who currently smoke at the time of pain consultation have been found to report higher pain scores.^21^ Although alcohol use affects pain thresholds,^22^ our results did not show an association between pain and current alcohol use. Our study is the first to identify a smoking component in reported pain following FFR, contributing new insights into the complex interplay between sociodemographic factors and perioperative pain experiences.

Although inpatient pain service consultation is often used for complex pain management, limited research has explored predictors of such consultations in patients undergoing HNC FFR. Research shows that most psychiatric conditions are associated with increased pain, with different pain thresholds depending on the specific psychiatric diagnosis.^23^ Moreover, prior work has found that psychiatric comorbidities and pain were associated with increased healthcare service use and may exhibit higher rates of consultations.^24^ Our findings support and extend this work by showing that MHD and preoperative narcotic prescription in the specific context of HNC FFR were independently associated with increased odds of inpatient pain consultation after controlling for inpatient psychiatry consultation. Additionally, we found that OCRFF predicted pain service consultation, suggesting that both patient‐ and surgery‐specific factors inform consult decisions. Importantly, the association between pain service consultation and higher pain scores should be interpreted as descriptive rather than causal, as the directionality (ie, reverse causation, with higher pain prompting consultation) cannot be determined. By identifying which patients are more likely to require additional pain management resources, our study provides actionable data to inform preoperative risk stratification and resource allocation for multidisciplinary pain care.

### Limitations

This study exhibits some limitations. We acknowledge the risk of incomplete information due to the retrospective chart review design. Other limitations of this study include its single‐institution design and the absence of prior head and neck surgery data. Thus, the patient cohort may not be representative of all FFR patients, including patients in other hospitals or regions of the country. The small sample size of non‐white patients in this cohort and the lack of understanding of potential underlying mechanisms (ie, provider biases and lower comfort level expressing true pain level) limit our analyses. To increase the sample size, we included non‐oncologic indications for FFR and accounted for this by controlling for FF indication in multivariable regressions and propensity matching; we also ran separate regressions with oncologic cases only. Regarding MHD, our definition underestimates the overall psychiatric comorbidity burden and does not capture the influence of all relevant mental health conditions (eg, substance use disorders) on postoperative pain outcomes. However, our focus on depression and anxiety diagnoses aimed to enhance interpretability and reduce heterogeneity that may arise from less common or more severe psychiatric diagnoses (eg, schizophrenia, bipolar disorder), which differ in symptom burden, treatment patterns, and pain perception.^25^ We used ICD and CPT codes to capture depression and anxiety in our patient population retrospectively, which only identified patients with previously treated or documented depression or anxiety. Since the PHQ‐9 and GAD‐7 tools are not always administered to our HNC patients, using standardized codes allowed us to reliably operationalize how we identified depression and anxiety. However, this likely underestimated the true prevalence of MHD in our patient population. Regarding pain measures, residual confounding related to prior pain experiences is possible. Chronic pain diagnoses encompass a heterogeneous set of conditions with variable pain severity. One‐time preoperative pain scores were recorded in fewer than half of cases, could not be verified as occurring before or after analgesic administration, and were not directly comparable to averaged postoperative pain scores across an entire day. Therefore, preoperative narcotic prescription was used as a surrogate measure of baseline pain burden, given its reliable coding. Additionally, the patient‐reported pain scale (0‐10) is inherently subjective. There may be unmeasured factors influencing reported postoperative pain that were not captured. However, we attempted to account for these by adjusting for a comprehensive set of demographic, clinical, and psychosocial variables as well as by examining both POD5 pain and pain trajectories over multiple postoperative days. Moreover, the threshold for a clinically significant difference in pain scores may be unclear; prior work suggests a pain intensity difference of 1.4 out of 10 may be clinically important.^26^ Although individual factors may not reach clinically important differences, compounding multiple significant factors increases this risk. Finally, associations involving pain consultation should be interpreted as descriptive rather than causal because pain consultation may serve as a surrogate marker of greater pain severity uncaptured by patient pain scores rather than as an independent exposure. Since institutional practice patterns and provider behavior influence pain consultation, consult utilization results do not necessarily reflect pain severity alone and may not generalize across institutions, warranting further investigation.

### Future Directions

The risk factors for postoperative pain in FFR identified in this study may inform proactive pain management strategies and preoperative connection to rehabilitation programs in patients who are more likely to experience high postoperative pain burden. Future studies should consider utilizing a prospective study design to ensure accurate and consistent data collection as well as collecting data from multiple institutions to obtain a more representative patient cohort. Research is needed to expand preliminary knowledge on the impact of sociodemographic factors on HNC FFR patient outcomes. Prior studies have shown that while demonstrated to be more effective in addressing postoperative pain, multimodal nonopioid analgesia (such as nonopioid pharmaceutical therapy, physical therapy, psychotherapy, and palliative care) is often underutilized in HNC patients.^27^, ^28^ In addition to future studies that improve risk stratification for pain burden, quality improvement studies are needed to assess the value of diversifying pain regimens typically prescribed for FF patients.

## Conclusion

This study is the first to provide a comprehensive assessment of the association between patient psychosocial characteristics and postoperative pain after FFR in a large cohort of 645 patients. Higher postoperative pain was independently associated with younger age, separated or divorced marital status, current smoking status, laryngeal site, OCRFFF, non‐oncologic indication, and MHD. Inpatient pain service consultation was more likely among patients with younger age, private insurance, OCRFFF, MHD, and preoperative narcotic prescription. These findings emphasize the importance of considering demographic, psychosocial, and clinical factors when anticipating pain trajectories and resource needs. A tailored, multidisciplinary approach to perioperative pain management, informed by these risk factors, may enhance postoperative recovery and long‐term outcomes in this complex patient population.

## Author Contributions

**Daniel R. S. Habib**, conceptualization, data curation, formal analysis, writing—original draft, writing—review and editing; **Sindhura Sridhar**, conceptualization, data curation, writing—review and editing; **Heezy Suh,** data curation, writing— original draft, writing—review and editing; **Daniel Larson,** data curation, writing—review and editing; **Brooke B. Swain,** data curation, writing—review and editing; **Kelly Vittetoe,** conceptualization, writing—review and editing; **Donald Sengstack,** data curation, writing—review and editing **Michael C. Topf**, supervision, conceptualization, writing—review and editing; **Melanie D. Hicks**, supervision, conceptualization, writing—review and editing.

## Disclosures

### Competing interests

The authors declare no conflicts of interest.

### Funding source

None.

## Supporting information

Supporting File
